# CDK4/6 Inhibitors in Patients Aged 80 and Older with HR+/HER2− Metastatic Breast Cancer: A Real-World Multicenter Study

**DOI:** 10.3390/cancers17203302

**Published:** 2025-10-13

**Authors:** Palma Fedele, Matteo Landriscina, Lucia Moraca, Antonio Cusmai, Antonio Gnoni, Antonella Licchetta, Laura Lanotte, Maria Nicla Pappagallo, Assunta Melaccio, Guido Giordano, Felicia Maria Maselli, Francesco Giuliani, Vincenzo Chiuri, Francesco Giotta, Federica Fumai, Gennaro Gadaleta-Caldarola

**Affiliations:** 1 Oncology Unit, “Dario Camberlingo” Hospital, Francavilla Fontana, 72021 Brindisi, Italy; federica.fumai@fermimonticelli.it; 2 U.O. Medical Oncology and Biomolecular Therapy, Department of Medical and Surgical Sciences, University of Foggia, 71100 Foggia, Italy; matteo.landriscina@unifg.it (M.L.); guido.giordano@unifg.it (G.G.); felicia.maselli@unifg.it (F.M.M.); 3 Oncology Unit, “Teresa Masselli Mascia” Hospital, San Severo, 71100 Foggia, Italy; lucia.moraca@aslfg.it; 4 Oncology Unit, I.R.C.C.S. “Giovanni Paolo II”, 70124 Bari, Italy; a.cusmai@oncologico.bari.it (A.C.); f.giotta@oncologico.bari.it (F.G.); 5 Oncology Unit, “Sacro Cuore di Gesù” Hospital, 73014 Gallipoli, Italy; antonio.gnoni@aslle.it (A.G.); antonella.licchetta@aslle.it (A.L.); vincenzo.chiuri@aslle.it (V.C.); 6 Oncology Unit, “Mons. A. R. Dimiccoli” Hospital, 70051 Barletta, Italy; laura.lanotte@aslbat.it (L.L.); gennaro.gadaleta@aslbat.it (G.G.-C.); 7 Oncology Unit, “San Paolo” Hospital, 70123 Bari, Italy; marianicla.pappagallo@asl.bari.it (M.N.P.); assunta.melaccio@asl.bari.it (A.M.); francesco.giuliani@asl.bari.it (F.G.)

**Keywords:** metastatic breast cancer, elderly patients, HR+/HER2− breast cancer, CDK4/6 inhibitors, real-world evidence, geriatric oncology, treatment tolerability

## Abstract

**Background:** Older adults aged ≥80 with HR+/HER2− metastatic breast cancer (MBC) are often underrepresented in clinical trials, leaving clinicians with limited data to guide treatment decisions. Given the increasing prevalence of this age group, real-world evidence is crucial to inform therapeutic strategies. **Methods:** We performed a multicenter retrospective study across seven Italian oncology units, focusing on patients aged 80 or above who received CDK4/6 inhibitors in combination with endocrine therapy between January 2020 and May 2024. Baseline characteristics, treatment details, and adverse events were recorded. Primary endpoints were progression-free survival (PFS) and overall survival (OS); toxicity was assessed using CTCAE v5.0. Follow-up was estimated using the reverse Kaplan–Meier method. **Results:** Eighty patients were included, with a median age of 83. Over half had visceral metastases, and 41.0% were frail (G8 ≤ 14). Approximately 44.0% of patients started treatment at a reduced dose. The median PFS was 13 months (95.0% CI 9.3–18.0), and the median OS was 15 months (95.0% CI 11.8–18.2). Hematologic toxicity was frequent, with neutropenia occurring in 58.8% (25.0% grade ≥ 3) and anemia in 12.5% (2.5% grade ≥ 3). Asthenia was reported in 16.2% (5.0% grade ≥ 3). Diarrhea occurred in 3.8% overall, mainly in patients treated with abemaciclib (42.9% any grade; 14.3% grade ≥ 3). ALT/AST elevations were observed in 8.8% (1.2% grade ≥ 3), and QTc prolongation in 2.5% (1.2% grade ≥ 3, all with ribociclib). Grade ≥ 3 events were uncommon and generally manageable. **Conclusions:** CDK4/6 inhibitor-based therapy is feasible in patients aged ≥80 years, with outcomes and tolerability comparable to those observed in younger elderly. Our results highlight the importance of individualized treatment strategies in this oldest-old population.

## Simple Summary

Older adults aged 80 years and above are rarely included in clinical trials evaluating cyclin-dependent kinase 4/6 (CDK4/6) inhibitors, leaving clinicians with little evidence to guide treatment decisions in this growing patient population. We conducted a multicenter, retrospective study across seven Italian oncology units to describe real-world outcomes of patients aged ≥80 years with HR+/HER2− metastatic breast cancer treated with CDK4/6 inhibitors plus endocrine therapy. Despite frailty and frequent comorbidities, treatment was feasible and generally well tolerated, with survival outcomes comparable to those reported in younger populations. These findings support individualized treatment strategies and suggest that advanced age alone should not preclude the use of CDK4/6 inhibitors

## 1. Introduction

In the past decade, treatment options for patients with metastatic hormone receptor-positive, HER2-negative (HR+/HER2−) breast cancer have expanded significantly, thanks especially to the integration of CDK4/6 inhibitors with endocrine therapy. This combination has not only changed routine clinical practice but has also become a cornerstone of first-line management, offering clear benefits in terms of disease control and, in many cases, overall survival [1,2,3,4,5,6,7,8].

Yet, while these advances have been widely embraced, they come with a notable caveat: the pivotal clinical trials that led to the approval of CDK4/6 inhibitors largely excluded older adults—particularly those aged 80 and above. Patients with frailty, multiple comorbidities, or reduced functional reserve were often not eligible, leaving a gap in evidence for precisely the population that is most rapidly growing in oncology clinics worldwide [9,10,11,12].

Today, individuals aged ≥80 years represent an increasingly relevant portion of the breast cancer population. Among them, HR+/HER2− tumors remain the most common subtype, accounting for approximately 75% of all breast cancers [13]. Despite this prevalence, older patients are frequently undertreated, often due to concerns about tolerability, drug interactions, or a perceived lack of benefit. This cautious approach may sometimes result in suboptimal care, as therapeutic decisions are guided more by chronological age than by a true assessment of physiological reserve or patient preferences [14].

Treating metastatic breast cancer in this age group involves a careful balancing act. On one hand, clinicians aim to provide effective systemic therapy; on the other, they must avoid excessive toxicity that could compromise quality of life. Dose reductions and close monitoring are common strategies, but without robust data, their impact remains uncertain. While some retrospective studies have begun to explore the role of CDK4/6 inhibitors in elderly patients with HR+/HER2− metastatic breast cancer [15], very few have focused specifically on the “oldest-old,” those aged 80 and above.

To help fill this gap, we conducted a multicenter, retrospective analysis across seven Italian oncology centers. Our study initially included patients aged 70 and over treated with CDK4/6 inhibitors in combination with endocrine therapy. From this broader dataset, we specifically examined the subgroup of 80 patients aged ≥80 years, aiming to shed light on treatment patterns, survival outcomes, and tolerability in this particularly vulnerable population. We also paid special attention to dose modifications and frailty assessments, seeking to understand how these factors influenced real-world practice and results.

## 2. Materials and Methods

This was a retrospective, multicenter observational study conducted across seven oncology units in Italy. We included patients aged 80 years or older who had a histologically confirmed diagnosis of hormone receptor-positive (HR+), HER2-negative (HER2−) metastatic breast cancer (MBC) and who started first line treatment with a CDK4/6 inhibitor between January 2020 and May 2024.

Patients were eligible if they began CDK4/6 inhibitor therapy in the metastatic setting, regardless of whether they had previously received treatment for early-stage disease. No specific exclusions were applied in terms of performance status or comorbidity burden, in order to reflect the clinical heterogeneity typical of real-world settings.

Data were retrospectively extracted from electronic medical records. We collected information on demographics (including age at treatment initiation and BMI), comorbid conditions, ECOG performance status, histologic subtype, and extent of disease (visceral vs. non-visceral). Treatment-related variables included the choice of endocrine partner (aromatase inhibitors or fulvestrant), the specific CDK4/6 inhibitor used (palbociclib, ribociclib, or abemaciclib), and whether therapy was initiated at a standard or reduced dose. Frailty was assessed using the G8 screening tool, a validated instrument commonly employed in geriatric oncology to identify patients at higher risk of poor outcomes. Exploratory subgroup analyses by G8 (≤14 vs. >14) were not conducted due to limited and non-harmonized patient-level time-to-event data across centers.

HER2 status was determined based on local pathology reports at the time of diagnosis and categorized simply as positive or negative, in accordance with diagnostic criteria available during the study period. No further distinction was made regarding HER2-low expression.

All CDK4/6 inhibitors were administered in line with local clinical practice. Treatment continued until radiographic or clinical progression, unacceptable toxicity, or death. None of the patients included in this study had previously received CDK4/6 inhibitors in the adjuvant setting.

The study was conducted in compliance with the European General Data Protection Regulation (GDPR, Regulation EU 2016/679) [16], and each participant was assigned an anonymized study ID. Ethical approval was granted by the Ethics Committee of the Puglia Region.

The primary endpoints of the study were progression-free survival (PFS) and overall survival (OS). PFS was defined as the time from treatment initiation to either disease progression or death from any cause, whichever occurred first. OS was defined as the time from initiation of CDK4/6 inhibitor therapy to death. Patients who had not experienced disease progression or death at the time of last follow-up were censored for survival analysis. Radiologic response assessments followed local clinical practice. When RECIST v1.1 evaluations were available in the medical record, they were used; otherwise, progression was determined per investigator.

Because of the retrospective, multicenter real-world design, follow-up was not protocol-mandated, and assessments followed routine clinical practice at each participating site. Clinical reviews were generally performed at treatment visits, while radiologic reassessments were performed at the treating physician’s discretion. The most frequent modality was contrast-enhanced CT of the chest/abdomen/pelvis; bone scan or PET/CT were obtained as clinically indicated (e.g., in patients with known or suspected bone involvement). Response and progression were abstracted from chart documentation.

Patients without progression or death were censored at the date of last disease assessment for PFS and at last known contact for OS.

Safety was assessed by reviewing all documented adverse events (AEs) that occurred during treatment. Events were graded according to the National Cancer Institute’s Common Terminology Criteria for Adverse Events (CTCAE), version 5.0 [17], and categorized by System Organ Class and Preferred Term using the MedDRA classification system, version 23.0. For each patient, only the highest grade of each adverse event type was reported.

Management of grade ≥ 3 adverse events generally followed product label guidance: CDK4/6 inhibitors were temporarily withheld until recovery to ≤grade 2, after which treatment was resumed at the same or a reduced dose at the discretion of the treating physician. Permanent discontinuation was reserved for recurrent or intolerable grade ≥ 3 events. Detailed data on the exact duration of treatment interruptions were not systematically captured in the medical records.

Descriptive statistics were used to summarize the cohort’s clinical and treatment characteristics. Continuous variables were reported as medians with ranges, while categorical variables were presented as absolute numbers and percentages. Survival analyses were performed using the Kaplan–Meier method, and follow-up duration was calculated using the reverse Kaplan–Meier technique. All statistical analyses were conducted using R software, version 4.4.1.

As this was a retrospective study, no formal sample size calculation was performed. The cohort size reflects the total number of eligible patients treated in the participating centers over the defined study period.

## 3. Results

A total of 80 patients aged 80 years or older with HR+/HER2− metastatic breast cancer were included in the final analysis. An overview of their demographic and clinical characteristics is presented in Table 1.

At the time of diagnosis, 68.7% of patients were at stage IV, and more than half (53.8%) had visceral metastases. Regarding tumor biology, the majority (60.0%) had luminal A subtype, while the remaining 40.0% had luminal B disease.

Comorbid conditions were common: only 10.2% of patients had no additional medical problems, whereas 68.7% had at least one comorbidity, and 21.3% had two or more. Body mass index (BMI) data showed that 41.3% of the cohort had a BMI under 25 kg/m^2^, while the remaining 58.7% were classified as overweight or obese (BMI ≥ 25 kg/m^2^).

Performance status was generally preserved; nearly all patients (97.5%) had an ECOG score of 1 or 2 at baseline. Nonetheless, frailty was frequently observed, with 41.2% of the cohort scoring ≤ 14 on the G8 scale.

Among the CDK4/6 inhibitors, palbociclib was the most frequently prescribed agent (62, 77.5%), followed by ribociclib (11, 13.8%) and abemaciclib (7, 8.8%).

**Figure 1:**
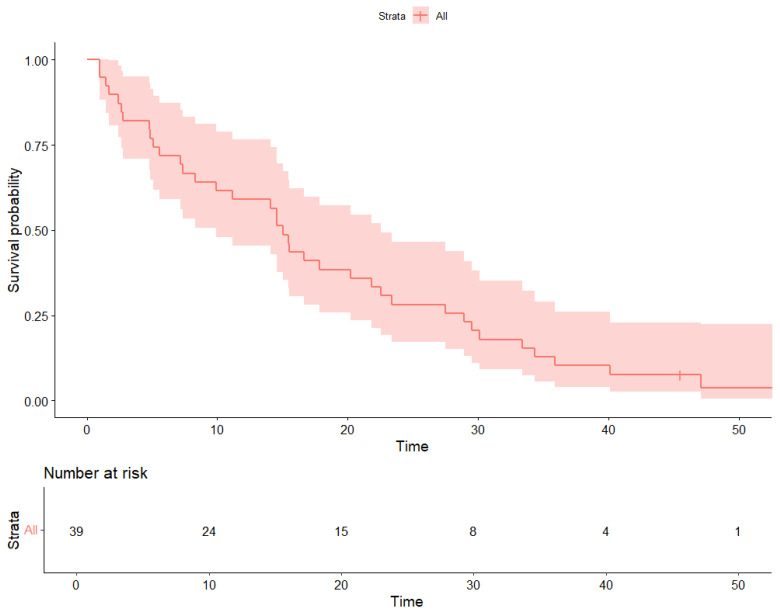
Progression-Free Survival (PFS).

**Figure 2:**
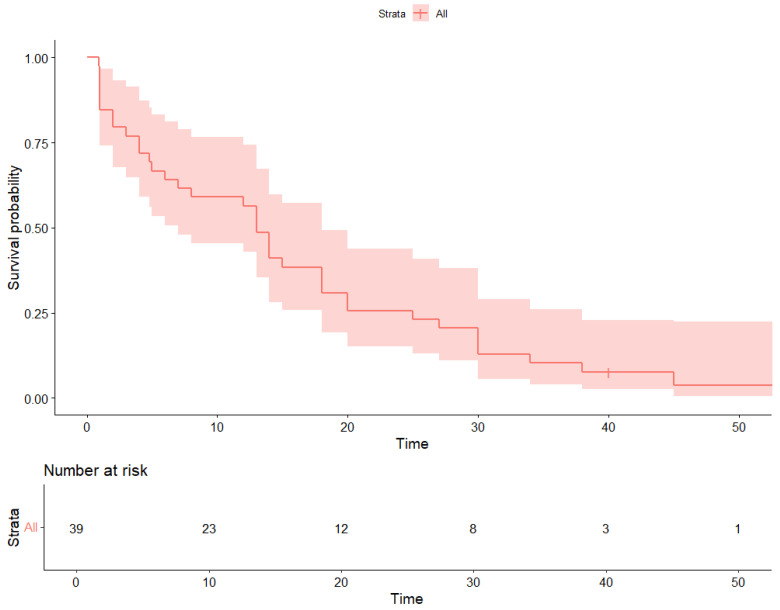
Overall Survival (OS).

Survival outcomes were estimated using Kaplan–Meier analysis. The median progression-free survival (PFS) was 13 months (95.0% CI 9.3–18.0) (Figure 1). At 12 months, approximately 52.0% of patients remained progression-free. Median overall survival (OS) was 15 months (95.0% CI 11.8–18.2) (Figure 2); survival rates were around 60.0% at 12 months and 30.0% at 24 months. The median follow-up for the entire cohort, calculated by the reverse Kaplan–Meier method, was 21 months (95% CI, 18–24).

Kaplan–Meier estimates of progression-free survival (PFS) in patients aged ≥80 years treated with CDK4/6 inhibitors.

Kaplan–Meier estimates of overall survival (OS) in patients aged ≥80 years treated with CDK4/6 inhibitors.

Regarding safety, the treatment was generally well tolerated (Table 2). Hematologic toxicities were the most frequently reported adverse events. Neutropenia occurred in 59.0% of patients, with 25.0% experiencing grade ≥ 3 events. Anemia was observed in 13.0% of cases, including 3.0% with grade ≥ 3 severity. Asthenia affected 16.0% of patients, with 5.0% experiencing grade ≥ 3 fatigue.

Diarrhea was more frequent among those treated with abemaciclib, occurring in 42.9% of patients, with 14.3% experiencing grade ≥ 3 events. In contrast, no cases were observed with palbociclib or ribociclib. Overall, diarrhea occurred in 3.8% of the cohort (1.2% grade ≥ 3).

QTc interval prolongation—typically associated with ribociclib—was observed in 9.0% of patients treated with this agent; one case reached grade ≥ 3 severity. In the overall cohort, QTc prolongation occurred in 3.0% (1.0% grade ≥ 3). Thrombocytopenia was reported in 4.0% of the cohort (1.0% grade ≥ 3), all limited to mild to moderate severity except one patient.

## 4. Discussion

This study offers novel insights into the treatment of metastatic HR+/HER2− breast cancer in the oldest-old population who are often overlooked in pivotal trials. Our findings suggest that, even in this frail and comorbid group, CDK4/6 inhibitors combined with endocrine therapy can offer clinically meaningful benefits. Notably, in these pivotal trials the proportion of patients aged ≥80 years was extremely limited (<2.0%), whereas our cohort exclusively included patients in this age group, addressing a critical evidence gap.

The median PFS of 13 months and OS of 15 months we observed are encouraging, especially considering the advanced age, the prevalence of comorbidities, and the high proportion of patients identified as frail using the G8 screening tool. These results point to the feasibility of maintaining disease control with systemic therapy when treatment is appropriately tailored. The comparatively shorter OS versus some real-world cohorts likely reflects case-mix differences—including a higher prevalence of visceral disease, the ≥80-year age threshold, a sizable use of reduced starting doses, and shorter follow-up—together with differences in supportive care.

Although over half of our cohort presented with visceral disease, we did not perform formal survival comparisons between visceral-only and visceral+bone subgroups due to sample size constraints. Such analyses would require larger datasets to ensure adequate statistical power and to draw reliable conclusions.

In terms of tolerability, the safety profile was consistent with previous reports, though with some differences reflective of this older population. Neutropenia remained the most frequent adverse event, affecting 59.0% of patients, with 25.0% experiencing it at grade ≥ 3. Anemia and asthenia were also observed but were mostly manageable. Diarrhea was more common with abemaciclib, as expected, but severe cases were rare. QTc prolongation and thrombocytopenia were infrequent and did not raise new safety concerns.

These observations align well with real-world experiences reported in other cohorts. The PALOMAGE study enrolled over 800 women aged ≥70 years treated with palbociclib plus endocrine therapy, reporting a median PFS of 18 months and confirming the relevance of geriatric screening tools such as G8 to guide treatment [18]. The IRE cohort similarly highlighted the feasibility of CDK4/6 inhibitors in older patients, with comparable PFS outcomes and an emphasis on individualized management []. The UK multicenter experience by Battisti et al. also supported the efficacy of these agents in older patients and showed that toxicity remained manageable when proactive monitoring was employed [19]. More recently, the PALMARES study compared palbociclib, ribociclib, and abemaciclib in a real-world population including patients ≥ 75 years, confirming similar outcomes across agents [20]. Likewise, the P-REALITY X study specifically examined patients aged ≥75 and reported meaningful clinical benefit with palbociclib [21]. Prospective studies such as POLARIS [22] and PROPSEA [23] further reinforced the safety and efficacy of CDK4/6 inhibitors in older adults, while a systematic review of palbociclib in older patients confirmed consistency across real-world and trial-based data [24].

A notable feature of our cohort is the high proportion of patients (43.6%) who started treatment at a reduced dose. This likely reflects a deliberate clinical choice aimed at minimizing toxicity in a population perceived as more vulnerable. While this individualized approach may have contributed to the relatively low incidence of high-grade adverse events [25], it raises the question of whether dose reductions affect efficacy. Because of the limited sample size and retrospective nature of the tudy, we did not perform formal survival comparisons between patients who started at full dose and those who received an initial dose reduction. Future prospective studies specifically designed to evaluate dose intensity and clinical outcomes in the oldest-old population would be useful to clarify this point.

This study has several strengths. It is, to our knowledge, one of the few real-world investigations specifically targeting patients aged ≥80 years receiving CDK4/6 inhibitors. Its multicenter nature enhances the generalizability of the findings, drawing from diverse clinical settings. Furthermore, incorporating the G8 frailty score offered a more detailed characterization of the patient population, beyond chronological age alone.

Of course, there are limitations. The retrospective design carries an inherent risk of selection bias and incomplete data. The relatively small sample size limited the possibility of performing multivariate analyses, which might have helped identify predictors of outcome. Additionally, frailty was assessed only through the G8 score; we lacked more detailed geriatric assessments such as cognitive function or social support, which could further refine patient stratification. In our cohort, detailed information on dose escalation and longitudinal dose intensity was not uniformly captured across centers, precluding an analysis of survival outcomes by starting dose or the rate of subsequent escalation. This represents an important limitation, as understanding the balance between tolerability and efficacy is particularly relevant in this vulnerable population

Moreover, the exact duration of treatment interruptions for grade ≥ 3 toxicities was not uniformly available across centers, precluding a precise quantification of dose intensity. In addition, the relatively small sample size precluded meaningful survival comparisons according to comorbidity burden.

Furthermore, data on treatment received after progression were not uniformly available across centers, preventing us from reporting the proportion of patients who were able to receive second-line therapy.

Despite these limitations, our results are consistent with broader real-world findings and support the notion that advanced age alone should not preclude the use of CDK4/6 inhibitors.

In randomized trials like PALOMA-2, MONALEESA-2, and MONARCH-2 [3,4,5,6,7,8], patients over 75 were markedly underrepresented—usually less than 10.0% of the study population. These studies also often excluded individuals with substantial comorbidities or frailty, limiting their applicability to everyday clinical practice. While these trials demonstrated longer median PFS (20–25 months), the outcomes may not be directly transferable to real-world oldest-old patients, who present with different clinical realities.

Our findings therefore serve as a complementary piece of evidence: in a more representative, real-world population aged ≥80 years, CDK4/6 inhibitors still show meaningful activity, and toxicity remains manageable—especially when proactive dose adjustments are employed. Real-world evidence like this is crucial to bridging the gap between clinical trials and routine care in geriatric oncology. As the population continues to age, future prospective studies specifically designed for ultra-elderly patients—complete with comprehensive geriatric assessments and stratified treatment approaches—will be essential to guide optimal, evidence-based care.

## 5. Conclusions

This real-world, multicenter study provides encouraging evidence that patients aged 80 and older with HR+/HER2− metastatic breast cancer can benefit from treatment with CDK4/6 inhibitors in combination with endocrine therapy. Despite the common presence of frailty and comorbid conditions, outcomes in terms of progression-free and overall survival were comparable to those seen in younger elderly populations.

## Figures and Tables

**Table 1:** Baseline Characteristics of the 80 Patients.

		n (%)
Stage at diagnosis	I–III	25 (31.3%)
	IV	55 (68.7%)
No. metastasis site	Soft Tissue/Bone	37 (46.2%)
	Visceral	43 (53.8%)
BC subtype	Luminal A	48 (60.0%)
	Luminal B	32 (40.0%)
Comorbidities	0	8 (10.0%)
	1	55 (68.7%)
	2 or more	17 (21.3%)
ECOG PS	0	2 (2.5%)
	1–2	78 (97.5%)
G8 score	>14	47 (58.8%)
	≤14	33 (41.2%)
Endocrine therapy	Letrozole/Anastrozole	41 (51.3%)
	Fulvestrant	39 (48.7%)
CDK4/6i	Abemaciclib	7 (8.8%)
	Palbociclib	62 (77.5%)
	Ribociclib	11 (13.7%)
Starting Dose	Standard	45 (56.4%)
	Reduced	35 (43.6%)
BMI	<25	33 (41.3%)
	≥25	47 (58.7%)

**Table 2:** Adverse Events by CDK4/6 Inhibitor.

AEs	Abemaciclib (N = 7) Any Grade	Ribociclib (N = 11) Any Grade	Palbociclib (N = 62) Any Grade	Abemaciclib (N = 7) Grade ≥ 3	Ribociclib (N = 11) Grade ≥ 3	Palbociclib (N = 62) Grade ≥ 3	All Patients (N = 80) Any Grade	All Patients (N = 80) Grade ≥ 3
Anemia	1 (14%)	1 (9%)	8 (13%)	1 (14%)	0 (0%)	1 (2%)	10 (13%)	2 (3%)
Neutropenia	2 (29%)	7 (64%)	38 (61%)	1 (14%)	2 (18%)	17 (27%)	47 (59%)	20 (25%)
Thrombocytopenia	0 (0%)	0 (0%)	3 (5%)	0 (0%)	0 (0%)	1 (2%)	3 (4%)	1 (1%)
Asthenia	1 (14%)	2 (18%)	10 (16%)	1 (14%)	1 (9%)	2 (3%)	13 (16%)	4 (5%)
Diarrhea	3 (43%)	0 (0%)	0 (0%)	1 (14%)	0 (0%)	0 (0%)	3 (4%)	1 (1%)
ALT/AST increased	1 (14%)	1 (9%)	5 (8%)	0 (0%)	0 (0%)	1 (2%)	7 (9%)	1 (1%)
QTc prolongation	0 (0%)	2 (18%)	0 (0%)	0 (0%)	1 (9%)	0 (0%)	2 (3%)	1 (1%)

## Data Availability

The data presented in this study are available on reasonable request from the corresponding author. The data are not publicly available due to privacy and ethical restrictions.
